# Crystal structure of (*E*)-4-{4-[eth­yl(2-hydroxy­eth­yl)amino]­styr­yl}-1-methyl­pyridinium nitrate hemihydrate

**DOI:** 10.1107/S2056989015000067

**Published:** 2015-01-14

**Authors:** Hui Zhang, Mu-Hua Peng, Xiao-Juan Wang, Xiao-Ying Li

**Affiliations:** a Department of Chemistry, Anhui University, Hefei 230039, People’s Republic of China; b Key Laboratory of Functional Inorganic Materials Chemistry, Hefei 230039, People’s Republic of China; c Department of Biology, Anhui University, Hefei 230039, People’s Republic of China; d Center for Stem Cell and Translational Medicine, School of Life Sciences, Hefei 230039, People’s Republic of China

**Keywords:** crystal structure, pyridinium derivative, hydrogen bonding, π–π stacking

## Abstract

The asymmetric unit of the title compound, C_18_H_23_N_2_O^+^·NO_3_^−^·0.5H_2_O, contains two independent 4-{4-[eth­yl(2-hy­droxy­eth­yl)amino]­styr­yl}-1-methyl­pyridin-1-ium cations, two nitrate anions and one lattice water mol­ecule. In the cations, the pyridine ring is twisted with respect by 7.98 (12) and 18.42 (10)° to the benzene ring. In the crystal, the cations, the anions and the lattice water mol­ecules are linked by O—H⋯O hydrogen bonds and weak C—H⋯O hydrogen bonds, forming a three-dimensional supra­molecular architecture. π–π stacking occurs between pyridine and benzene rings of adjacent cations, the centroid–centroid distances being 3.8169 (15) and 3.8663 (14) Å. In the crystal, one of the independent cations is disordered, the central vinyl unit and the terminal hy­droxy­lethyl group being disordered over two sets of sites with site occupancy factors of 0.600 (6) and 0.400 (6).

## Related literature   

For applications of related pyridinium derivatives, see: Marder *et al.* (1994 ▸); Yang *et al.* (2013 ▸).
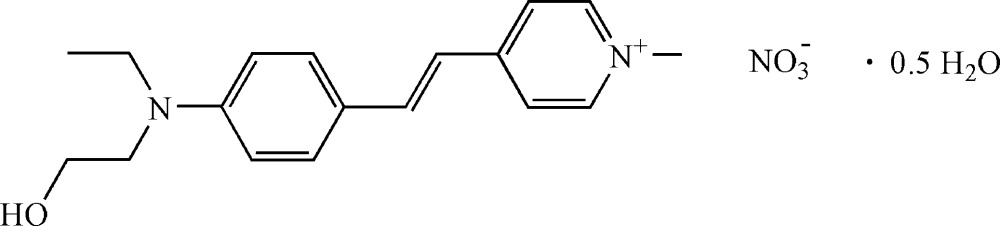


## Experimental   

### Crystal data  

C_18_H_23_N_2_O^+^·NO_3_^−^·0.5H_2_O
*M* *_r_* = 354.40
Monoclinic, 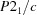

*a* = 15.5115 (17) Å
*b* = 14.6022 (16) Å
*c* = 16.4765 (19) Å
β = 101.835 (1)°
*V* = 3652.6 (7) Å^3^
*Z* = 8
Mo *K*α radiation
μ = 0.09 mm^−1^
*T* = 296 K
0.23 × 0.22 × 0.21 mm

### Data collection  

Bruker APEXII CCD area-detector diffractometer
26119 measured reflections
6583 independent reflections
4645 reflections with *I* > 2σ(*I*)
*R*_int_ = 0.027

### Refinement  

*R*[*F* ^2^ > 2σ(*F* ^2^)] = 0.057
*wR*(*F* ^2^) = 0.177
*S* = 1.10
6583 reflections
511 parameters
2 restraints
H-atom parameters constrained
Δρ_max_ = 0.46 e Å^−3^
Δρ_min_ = −0.22 e Å^−3^

### 

Data collection: *APEX2* (Bruker, 2007 ▸); cell refinement: *SAINT* (Bruker, 2007 ▸); data reduction: *SAINT*; program(s) used to solve structure: *SHELXTL* (Sheldrick, 2008 ▸); program(s) used to refine structure: *SHELXTL*; molecular graphics: *SHELXTL*; software used to prepare material for publication: *SHELXTL*.

## Supplementary Material

Crystal structure: contains datablock(s) I, Global. DOI: 10.1107/S2056989015000067/xu5834sup1.cif

Structure factors: contains datablock(s) I. DOI: 10.1107/S2056989015000067/xu5834Isup2.hkl

Supporting information file. DOI: 10.1107/S2056989015000067/xu5834Isup3.cml

**Fig. 1.**
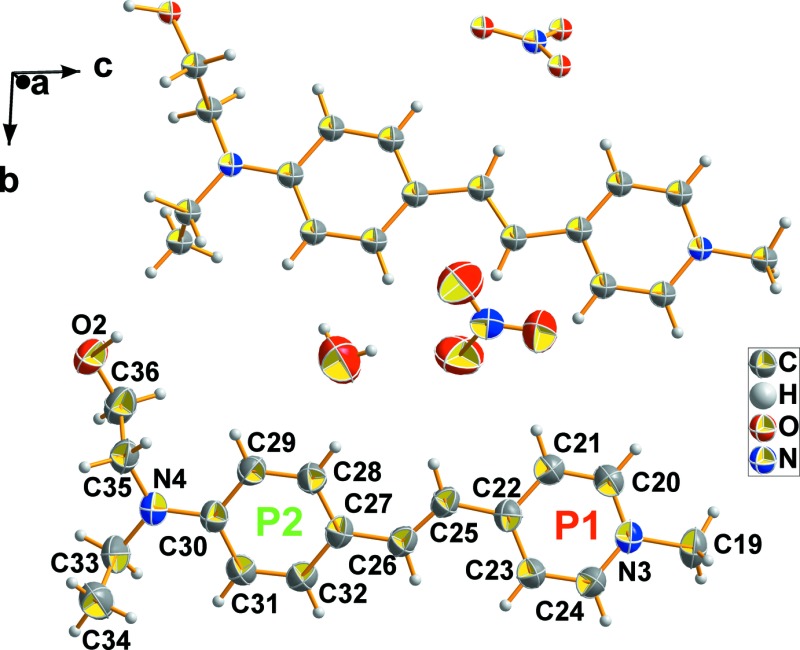
: The structure of the title compound, with atom labels and 50% probability displacement ellipsoids for non-H atoms.

. DOI: 10.1107/S2056989015000067/xu5834fig1.tif
The structure of the title compound, with atom labels and 50% probability displacement ellipsoids for non-H atoms.

CCDC reference: 1042027

Additional supporting information:  crystallographic information; 3D view; checkCIF report

## Figures and Tables

**Table 1:** Hydrogen-bond geometry (, )

*D*H*A*	*D*H	H*A*	*D* *A*	*D*H*A*
O1H1*E*O2^i^	0.96	1.83	2.719(10)	153
O2H2*C*O6^ii^	0.96	2.17	2.939(4)	137
O2H2*C*O7^ii^	0.96	2.38	3.242(3)	150
O9H9*B*O3	0.92	2.15	2.988(4)	151
O9H9*B*O4	0.92	2.43	3.214(4)	142
O9H9*D*O7^ii^	0.94	2.28	3.212(4)	170
C1H1*B*O4^iii^	0.96	2.56	3.480(4)	159
C2H2O4^iii^	0.93	2.54	3.380(3)	151
C6H6O5^iv^	0.93	2.47	3.204(4)	136
C15H15*B*O7^ii^	0.97	2.55	3.504(4)	168
C19H19*A*O8^iv^	0.96	2.48	3.166(3)	129
C20H20O5^iv^	0.93	2.47	3.348(3)	156
C24H24O9^v^	0.93	2.58	3.215(4)	126
C33H33*A*O1^vi^	0.97	2.53	3.400(10)	149

Symmetry codes: (i) 
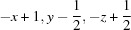
; (ii) 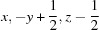; (iii) 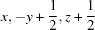; (iv) 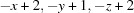; (v) 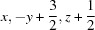; (vi) 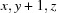.

## supplementary crystallographic information

### S1. Comment

Pyridiniums are important kind of nonlinear optical materials (Marder *et al.*, 1994), because their strong intramolecular charge transfer capability and high thermal stability. Thus organic salts have been employed in applications cover various fields, including frequency up-conversion, photorefraction, distinct two-photon absorption and fluorescent probe (Yang *et al.*, 2013).

In this paper, a novel pyridinium derivative was synthesized (Fig. 1). The asymmetric unit of the title compound contains two independent 4-(4-(ethyl(2-hydroxyethyl)amino)styryl)-1-methylpyridin-1-ium cations, two nitrate anions and one lattice water molecule. In the cations, the pyridine ring is twisted with respect to the benzene ring at 7.98 (12) and 18.42 (10)°, respectively. In the crystal, the cations, the anions and the lattice water molecules are linked by O—H···O hydrogen bonds and weak C—H···O hydrogen bonds (Table 1), forming the three dimensional supramolecular architecture. π-π stacking occurs between pyridine and benzene rings of adjacent cations, centroid-to-centroid distances being 3.8169 (15) and 3.8663 (14) Å. In the crystal, one of two independent cations is disordered, the central vinyl unit and the terminal hydroxylethyl group are disordered over two positions with a site occupancy factor ratio of 0.609 (4):0.391 (4).

### S2. Experimental

The intermediate 1,4-dimethylpyridin-1-ium was synthesized by mixing 4-methylpyridine (6.20 g, 65 mmol) with iodomethane (14.40 g, 100 mmol) which was pre-dissolved in ethanol (10 ml). The mixture was heated to reflux for 20 min and then cooled to room temperature. White crystal (14.64 g, 96%) was obtained after filtered and washed with ethanol for 3 times. The intermediate 4-(ethyl(2-hydroxyethyl)amino)benzaldehyde was synthesized based on 2-(ethyl(phenyl)amino)ethanol(12.40 g, 75 mmol), phosphorus oxychloride (35.30 g, 230 mmol) and DMF (8.40 g, 115 mmol) *via* vilsmeier reaction.

The title compound was synthesized by mixing 4-(ethyl(2-hydroxyethyl)amino)benzaldehyde (1.90 g, 10 mmol) with 1,4-dimethylpyridin-1-ium (2.40 g, 10 mmol) in ethanol (30 ml), and then piperidine (0.1 ml, 1 mmol) was injected. The mixture was heated to reflux for 1 d and then cooled to room temperature. AgNO_3_ (1.70 g, 10 mmol) pre-dissolved in 30 ml ethanol was instilled into the former mixture, and heated to reflux for 2 h. The precipitate was filtered and washed with ethanol for 3 times. Red crystal (2.18 g, 63%) was obtained after recrystallized in DCM. ^1^H NMR: (400 Hz, DMSO-d_6_), d(p.p.m.): 8.66 (d, 2H), 8.02 (d, 2H), 7.89 (d, 1H), 7.56 (d, 2H), 7.12 (d, 1H), 6.78 (d, 2H), 4.80 (s, 1H), 4.16 (s, 3H), 3.51 (m, 6H), 1.12 (t, 3H).

### S3. Refinement

The water H atoms were located in a difference Fourier map and ridden on its parent atom with *U*_iso_(H) = 1.5*U*_eq_(O). Other H atoms were placed in geometrically idealized positions (C—H = 0.93–0.97 Å) and allowed to ride on their parent atoms with *U*_iso_(H) = 1.5*U*_eq_(C) for the methyl H atoms and 1.2*U*_eq_(C) for the others.

### Crystal data

**Table:**

C_18_H_23_N_2_O^+^·NO_3_^−^·0.5H_2_O	*F*(000) = 1512
*M**_r_* = 354.40	*D*_x_ = 1.289 Mg m^−^^3^
Monoclinic, *P*2_1_/*c*	Melting point: 395 K
Hall symbol: -P 2ybc	Mo *K*α radiation, λ = 0.71073 Å
*a* = 15.5115 (17) Å	Cell parameters from 7118 reflections
*b* = 14.6022 (16) Å	θ = 2.4–24.1°
*c* = 16.4765 (19) Å	µ = 0.09 mm^−^^1^
β = 101.835 (1)°	*T* = 296 K
*V* = 3652.6 (7) Å^3^	Block, red
*Z* = 8	0.23 × 0.22 × 0.21 mm

### Data collection

**Table:**

Bruker APEXII CCD area-detector diffractometer	4645 reflections with *I* > 2σ(*I*)
Radiation source: fine-focus sealed tube	*R*_int_ = 0.027
Graphite monochromator	θ_max_ = 25.2°, θ_min_ = 1.9°
phi and ω scans	*h* = −18→18
26119 measured reflections	*k* = −17→16
6583 independent reflections	*l* = −19→19

### Refinement

**Table:**

Refinement on *F*^2^	Secondary atom site location: difference Fourier map
Least-squares matrix: full	Hydrogen site location: inferred from neighbouring sites
*R*[*F*^2^ > 2σ(*F*^2^)] = 0.057	H-atom parameters constrained
*wR*(*F*^2^) = 0.177	*w* = 1/[σ^2^(*F*_o_^2^) + (0.0857*P*)^2^ + 0.8929*P*] where *P* = (*F*_o_^2^ + 2*F*_c_^2^)/3
*S* = 1.10	(Δ/σ)_max_ < 0.001
6583 reflections	Δρ_max_ = 0.46 e Å^−^^3^
511 parameters	Δρ_min_ = −0.22 e Å^−^^3^
2 restraints	Extinction correction: *SHELXL*, Fc^*^=kFc[1+0.001xFc^2^λ^3^/sin(2θ)]^-1/4^
Primary atom site location: structure-invariant direct methods	Extinction coefficient: 0.0028 (7)

### Special details

**Table:**

Geometry. All esds (except the esd in the dihedral angle between two l.s. planes) are estimated using the full covariance matrix. The cell esds are taken into account individually in the estimation of esds in distances, angles and torsion angles; correlations between esds in cell parameters are only used when they are defined by crystal symmetry. An approximate (isotropic) treatment of cell esds is used for estimating esds involving l.s. planes.
Refinement. Refinement of *F*^2^ against ALL reflections. The weighted *R*-factor *wR* and goodness of fit *S* are based on *F*^2^, conventional *R*-factors *R* are based on *F*, with *F* set to zero for negative *F*^2^. The threshold expression of *F*^2^ > σ(*F*^2^) is used only for calculating *R*-factors(gt) *etc*. and is not relevant to the choice of reflections for refinement. *R*-factors based on *F*^2^ are statistically about twice as large as those based on *F*, and *R*- factors based on ALL data will be even larger.

### Fractional atomic coordinates and isotropic or equivalent isotropic displacement parameters (Å^2^)

**Table:**

	*x*	*y*	*z*	*U*_iso_*/*U*_eq_	Occ. (<1)
C1	0.90771 (18)	0.3566 (2)	1.20540 (14)	0.0768 (7)	
H1A	0.9608	0.3922	1.2134	0.115*	
H1B	0.9203	0.2978	1.2312	0.115*	
H1C	0.8648	0.3877	1.2299	0.115*	
C2	0.85027 (15)	0.26123 (17)	1.08554 (14)	0.0615 (6)	
H2	0.8578	0.2106	1.1205	0.074*	
C3	0.81616 (17)	0.2502 (2)	1.00297 (17)	0.0752 (7)	
H3	0.8004	0.1918	0.9827	0.090*	
C4	0.80435 (15)	0.3235 (3)	0.94863 (15)	0.0805 (9)	
C5	0.83069 (19)	0.4071 (2)	0.98344 (17)	0.0849 (9)	
H5	0.8253	0.4587	0.9497	0.102*	
C6	0.86415 (17)	0.41630 (18)	1.06533 (16)	0.0707 (7)	
H6	0.8815	0.4739	1.0867	0.085*	
C7	0.7703 (2)	0.3399 (3)	0.8587 (3)	0.0563 (12)	0.600 (6)
H7	0.7737	0.3971	0.8348	0.068*	0.600 (6)
C8	0.7347 (3)	0.2683 (3)	0.8143 (3)	0.0559 (12)	0.600 (6)
H8	0.7331	0.2111	0.8387	0.067*	0.600 (6)
C7'	0.7618 (4)	0.2755 (5)	0.8675 (4)	0.0472 (15)	0.400 (6)
H7'	0.7491	0.2132	0.8646	0.057*	0.400 (6)
C8'	0.7444 (4)	0.3302 (5)	0.8016 (3)	0.0445 (15)	0.400 (6)
H8'	0.7598	0.3918	0.8032	0.053*	0.400 (6)
C9	0.69711 (17)	0.2822 (3)	0.72359 (16)	0.0918 (11)	
C10	0.66142 (18)	0.2018 (3)	0.69138 (16)	0.0870 (9)	
H10	0.6657	0.1513	0.7264	0.104*	
C11	0.61969 (17)	0.1909 (2)	0.61056 (15)	0.0768 (7)	
H11	0.5962	0.1341	0.5925	0.092*	
C12	0.61162 (15)	0.26392 (17)	0.55440 (13)	0.0611 (6)	
C13	0.64847 (15)	0.34740 (18)	0.58505 (15)	0.0668 (6)	
H13	0.6452	0.3978	0.5501	0.080*	
C14	0.68982 (15)	0.3550 (2)	0.66746 (18)	0.0845 (9)	
H14	0.7140	0.4113	0.6865	0.101*	
C15	0.55776 (18)	0.32915 (19)	0.41559 (15)	0.0742 (7)	
H15A	0.5491	0.3052	0.3596	0.089*	
H15B	0.6109	0.3660	0.4253	0.089*	
C16	0.4805 (2)	0.3897 (2)	0.4224 (2)	0.1008 (10)	
H16A	0.4272	0.3542	0.4108	0.151*	
H16B	0.4763	0.4389	0.3832	0.151*	
H16C	0.4889	0.4143	0.4775	0.151*	
C17	0.5069 (3)	0.1801 (3)	0.4512 (3)	0.0483 (11)	0.600 (6)
H17A	0.4569	0.2018	0.4104	0.058*	0.600 (6)
H17B	0.4859	0.1604	0.4999	0.058*	0.600 (6)
C18	0.5512 (4)	0.0980 (4)	0.4149 (4)	0.0711 (16)	0.600 (6)
H18A	0.5754	0.1179	0.3680	0.085*	0.600 (6)
H18B	0.5986	0.0729	0.4567	0.085*	0.600 (6)
O1	0.4828 (8)	0.0285 (7)	0.3885 (6)	0.112 (4)	0.600 (6)
H1E	0.4735	0.0190	0.3297	0.167*	0.600 (6)
C18'	0.4877 (6)	0.1240 (10)	0.4312 (6)	0.107 (4)	0.400 (6)
H18C	0.4439	0.1631	0.3974	0.128*	0.400 (6)
H18D	0.4784	0.1216	0.4876	0.128*	0.400 (6)
C17'	0.5706 (7)	0.1467 (9)	0.4270 (6)	0.089 (3)	0.400 (6)
H17C	0.5790	0.1506	0.3704	0.106*	0.400 (6)
H17D	0.6138	0.1056	0.4588	0.106*	0.400 (6)
O1'	0.4930 (10)	0.0277 (8)	0.3927 (5)	0.076 (4)	0.400 (6)
H1'2	0.4364	0.0019	0.3696	0.114*	0.400 (6)
C19	0.99709 (15)	0.79038 (18)	1.10168 (12)	0.0637 (6)	
H19A	1.0595	0.8004	1.1103	0.096*	
H19B	0.9858	0.7369	1.1317	0.096*	
H19C	0.9696	0.8426	1.1212	0.096*	
C20	0.94022 (14)	0.69243 (15)	0.98244 (13)	0.0542 (5)	
H20	0.9492	0.6424	1.0182	0.065*	
C21	0.90635 (14)	0.67910 (15)	0.90044 (13)	0.0526 (5)	
H21	0.8929	0.6200	0.8808	0.063*	
C22	0.89132 (12)	0.75282 (14)	0.84503 (12)	0.0453 (5)	
C23	0.91638 (14)	0.83908 (15)	0.87844 (12)	0.0526 (5)	
H23	0.9098	0.8901	0.8440	0.063*	
C24	0.95045 (14)	0.84912 (15)	0.96132 (13)	0.0551 (5)	
H24	0.9666	0.9071	0.9824	0.066*	
C25	0.85149 (13)	0.73746 (14)	0.75868 (12)	0.0478 (5)	
H25	0.8487	0.6776	0.7391	0.057*	
C26	0.81846 (13)	0.80381 (15)	0.70526 (12)	0.0491 (5)	
H26	0.8241	0.8632	0.7261	0.059*	
C27	0.77504 (13)	0.79460 (14)	0.61922 (12)	0.0471 (5)	
C28	0.76679 (13)	0.71118 (15)	0.57584 (12)	0.0495 (5)	
H28	0.7903	0.6584	0.6035	0.059*	
C29	0.72528 (13)	0.70496 (14)	0.49425 (12)	0.0500 (5)	
H29	0.7217	0.6484	0.4678	0.060*	
C30	0.68793 (12)	0.78265 (14)	0.44965 (12)	0.0476 (5)	
C31	0.69637 (14)	0.86600 (15)	0.49283 (13)	0.0537 (5)	
H31	0.6733	0.9191	0.4655	0.064*	
C32	0.73814 (14)	0.87069 (15)	0.57474 (13)	0.0531 (5)	
H32	0.7418	0.9271	0.6015	0.064*	
C33	0.59927 (15)	0.85546 (17)	0.32473 (14)	0.0644 (6)	
H33A	0.5737	0.8915	0.3633	0.077*	
H33B	0.5515	0.8341	0.2813	0.077*	
C34	0.65901 (19)	0.9161 (2)	0.28658 (17)	0.0828 (8)	
H34A	0.7080	0.9353	0.3287	0.124*	
H34B	0.6268	0.9690	0.2624	0.124*	
H34C	0.6802	0.8827	0.2444	0.124*	
C35	0.64478 (14)	0.69331 (16)	0.32029 (13)	0.0564 (5)	
H35A	0.7005	0.6621	0.3392	0.068*	
H35B	0.6407	0.7096	0.2625	0.068*	
C36	0.57032 (16)	0.62827 (18)	0.32676 (15)	0.0687 (7)	
H36A	0.5748	0.6106	0.3842	0.082*	
H36B	0.5144	0.6592	0.3083	0.082*	
N1	0.87290 (11)	0.34430 (13)	1.11629 (10)	0.0523 (4)	
N2	0.56995 (16)	0.25335 (15)	0.47325 (12)	0.0787 (7)	
N3	0.96098 (11)	0.77709 (12)	1.01264 (10)	0.0490 (4)	
N4	0.64398 (12)	0.77638 (13)	0.36832 (10)	0.0572 (5)	
O2	0.57310 (12)	0.54893 (12)	0.27801 (11)	0.0821 (6)	
H2C	0.6205	0.5104	0.3050	0.123*	
N5	0.93868 (15)	0.44156 (15)	0.78940 (13)	0.0682 (6)	
N6	0.78332 (16)	0.02985 (13)	0.87218 (15)	0.0672 (6)	
O8	0.85952 (15)	0.05457 (16)	0.89505 (17)	0.1168 (8)	
O4	0.94309 (18)	0.37594 (17)	0.74487 (15)	0.1177 (8)	
O7	0.73564 (19)	0.02154 (17)	0.92070 (18)	0.1231 (9)	
O5	0.9769 (2)	0.44806 (18)	0.86029 (15)	0.1382 (11)	
O3	0.88408 (18)	0.50073 (15)	0.76188 (17)	0.1210 (8)	
O6	0.75723 (19)	0.01095 (17)	0.79874 (15)	0.1199 (8)	
O9	0.8905 (2)	0.5013 (2)	0.58187 (15)	0.1366 (10)	
H9B	0.8821	0.4800	0.6325	0.205*	
H9D	0.8405	0.4921	0.5391	0.205*	

### Atomic displacement parameters (Å^2^)

**Table:**

	*U*^11^	*U*^22^	*U*^33^	*U*^12^	*U*^13^	*U*^23^
C1	0.0786 (16)	0.106 (2)	0.0413 (12)	−0.0050 (15)	0.0022 (11)	−0.0084 (13)
C2	0.0641 (14)	0.0629 (15)	0.0585 (14)	−0.0034 (11)	0.0151 (11)	0.0002 (11)
C3	0.0646 (15)	0.0921 (19)	0.0691 (17)	−0.0109 (14)	0.0140 (12)	−0.0327 (15)
C4	0.0485 (13)	0.150 (3)	0.0414 (13)	0.0201 (16)	0.0046 (10)	−0.0071 (16)
C5	0.0829 (18)	0.109 (2)	0.0580 (16)	0.0209 (17)	0.0048 (13)	0.0295 (16)
C6	0.0754 (16)	0.0599 (15)	0.0729 (16)	0.0041 (12)	0.0062 (12)	0.0064 (12)
C7	0.061 (2)	0.054 (3)	0.052 (3)	0.0050 (19)	0.008 (2)	0.004 (2)
C8	0.055 (2)	0.056 (3)	0.056 (3)	0.0024 (19)	0.010 (2)	0.002 (2)
C7'	0.054 (3)	0.045 (4)	0.039 (4)	0.000 (3)	0.000 (3)	−0.004 (3)
C8'	0.049 (3)	0.047 (4)	0.035 (4)	0.001 (3)	0.003 (2)	−0.005 (3)
C9	0.0524 (15)	0.173 (3)	0.0453 (14)	0.0224 (18)	−0.0006 (11)	−0.0187 (19)
C10	0.0720 (17)	0.135 (3)	0.0489 (14)	0.0127 (17)	0.0000 (12)	0.0131 (16)
C11	0.0777 (16)	0.0890 (19)	0.0570 (14)	−0.0100 (14)	−0.0019 (12)	0.0097 (13)
C12	0.0575 (13)	0.0739 (16)	0.0470 (12)	−0.0100 (11)	−0.0012 (10)	−0.0030 (11)
C13	0.0566 (13)	0.0791 (17)	0.0615 (14)	−0.0049 (12)	0.0046 (11)	−0.0168 (12)
C14	0.0482 (13)	0.123 (3)	0.0783 (19)	−0.0032 (14)	0.0047 (12)	−0.0551 (19)
C15	0.0845 (18)	0.0842 (18)	0.0494 (13)	−0.0191 (15)	0.0035 (12)	0.0058 (12)
C16	0.093 (2)	0.117 (3)	0.087 (2)	0.0015 (19)	0.0076 (17)	0.0161 (19)
C17	0.048 (2)	0.047 (2)	0.046 (2)	−0.0116 (17)	0.0010 (16)	−0.0128 (17)
C18	0.072 (4)	0.056 (3)	0.083 (3)	−0.009 (3)	0.011 (3)	−0.030 (3)
O1	0.132 (6)	0.071 (6)	0.125 (6)	−0.039 (5)	0.010 (5)	−0.026 (4)
C18'	0.076 (6)	0.173 (13)	0.074 (6)	0.018 (8)	0.019 (5)	0.027 (7)
C17'	0.084 (6)	0.125 (9)	0.060 (5)	0.014 (6)	0.024 (4)	0.001 (5)
O1'	0.135 (9)	0.053 (7)	0.027 (3)	0.006 (6)	−0.011 (4)	−0.003 (3)
C19	0.0610 (13)	0.0891 (17)	0.0378 (11)	−0.0066 (12)	0.0027 (9)	−0.0020 (11)
C20	0.0587 (12)	0.0544 (13)	0.0489 (12)	0.0007 (10)	0.0097 (10)	0.0067 (10)
C21	0.0598 (12)	0.0481 (12)	0.0493 (12)	−0.0021 (9)	0.0094 (9)	−0.0014 (9)
C22	0.0443 (10)	0.0515 (12)	0.0421 (10)	0.0020 (8)	0.0135 (8)	−0.0019 (9)
C23	0.0647 (13)	0.0490 (12)	0.0444 (11)	−0.0019 (10)	0.0119 (9)	0.0017 (9)
C24	0.0644 (13)	0.0525 (13)	0.0478 (12)	−0.0047 (10)	0.0104 (10)	−0.0048 (10)
C25	0.0517 (11)	0.0515 (12)	0.0409 (11)	−0.0009 (9)	0.0116 (8)	−0.0063 (9)
C26	0.0522 (11)	0.0527 (12)	0.0432 (11)	−0.0019 (9)	0.0116 (9)	−0.0063 (9)
C27	0.0452 (10)	0.0548 (12)	0.0413 (10)	−0.0006 (9)	0.0089 (8)	−0.0029 (9)
C28	0.0493 (11)	0.0518 (12)	0.0458 (11)	0.0028 (9)	0.0063 (9)	0.0012 (9)
C29	0.0514 (11)	0.0491 (12)	0.0478 (11)	−0.0025 (9)	0.0062 (9)	−0.0058 (9)
C30	0.0439 (10)	0.0539 (12)	0.0440 (11)	−0.0029 (9)	0.0067 (8)	−0.0008 (9)
C31	0.0589 (12)	0.0506 (12)	0.0493 (12)	0.0049 (10)	0.0058 (9)	0.0040 (9)
C32	0.0595 (12)	0.0491 (12)	0.0498 (12)	0.0011 (10)	0.0095 (9)	−0.0051 (9)
C33	0.0611 (13)	0.0721 (16)	0.0528 (13)	0.0033 (11)	−0.0051 (10)	0.0043 (11)
C34	0.0906 (19)	0.0825 (19)	0.0718 (17)	−0.0043 (15)	0.0082 (14)	0.0177 (14)
C35	0.0587 (13)	0.0676 (14)	0.0415 (11)	−0.0068 (10)	0.0072 (9)	−0.0032 (10)
C36	0.0617 (14)	0.0813 (17)	0.0605 (14)	−0.0140 (12)	0.0068 (11)	−0.0096 (12)
N1	0.0501 (10)	0.0636 (12)	0.0421 (9)	0.0016 (8)	0.0067 (7)	−0.0010 (8)
N2	0.1049 (16)	0.0694 (13)	0.0487 (11)	−0.0279 (12)	−0.0153 (10)	0.0027 (10)
N3	0.0475 (9)	0.0600 (11)	0.0391 (9)	−0.0025 (8)	0.0074 (7)	−0.0001 (8)
N4	0.0635 (11)	0.0592 (11)	0.0439 (10)	−0.0003 (9)	−0.0003 (8)	−0.0005 (8)
O2	0.0864 (12)	0.0730 (12)	0.0745 (11)	−0.0102 (9)	−0.0126 (9)	−0.0143 (9)
N5	0.0845 (14)	0.0557 (13)	0.0615 (13)	−0.0127 (11)	0.0085 (11)	0.0031 (10)
N6	0.0795 (15)	0.0393 (10)	0.0782 (15)	0.0004 (10)	0.0051 (12)	−0.0024 (10)
O8	0.0925 (16)	0.0859 (15)	0.162 (2)	−0.0189 (12)	0.0020 (15)	−0.0141 (15)
O4	0.160 (2)	0.0937 (17)	0.1067 (17)	0.0182 (15)	0.0434 (16)	−0.0263 (14)
O7	0.130 (2)	0.1003 (18)	0.157 (2)	−0.0055 (15)	0.0733 (19)	−0.0217 (16)
O5	0.187 (3)	0.1163 (19)	0.0826 (15)	−0.0502 (18)	−0.0385 (16)	0.0145 (13)
O3	0.135 (2)	0.0740 (14)	0.144 (2)	0.0231 (14)	0.0054 (16)	0.0058 (14)
O6	0.165 (2)	0.0971 (17)	0.0860 (16)	−0.0138 (15)	−0.0022 (15)	0.0021 (13)
O9	0.161 (2)	0.142 (2)	0.0993 (17)	−0.0210 (19)	0.0081 (16)	0.0324 (16)

### Geometric parameters (Å, º)

**Table:**

C1—N1	1.468 (3)	C17'—H17D	0.9700
C1—H1A	0.9600	O1'—H1E	1.0274
C1—H1B	0.9600	O1'—H1'2	0.9600
C1—H1C	0.9600	C19—N3	1.472 (3)
C2—N1	1.334 (3)	C19—H19A	0.9600
C2—C3	1.364 (3)	C19—H19B	0.9600
C2—H2	0.9300	C19—H19C	0.9600
C3—C4	1.383 (4)	C20—N3	1.347 (3)
C3—H3	0.9300	C20—C21	1.359 (3)
C4—C5	1.376 (4)	C20—H20	0.9300
C4—C7	1.488 (6)	C21—C22	1.400 (3)
C4—C7'	1.533 (8)	C21—H21	0.9300
C5—C6	1.349 (4)	C22—C23	1.397 (3)
C5—H5	0.9300	C22—C25	1.448 (3)
C6—N1	1.335 (3)	C23—C24	1.367 (3)
C6—H6	0.9300	C23—H23	0.9300
C7—C8	1.330 (2)	C24—N3	1.338 (3)
C7—H7	0.9300	C24—H24	0.9300
C8—C9	1.502 (6)	C25—C26	1.338 (3)
C8—H8	0.9300	C25—H25	0.9300
C7'—C8'	1.331 (2)	C26—C27	1.446 (3)
C7'—H7'	0.9300	C26—H26	0.9300
C8'—C9	1.514 (7)	C27—C32	1.388 (3)
C8'—H8'	0.9300	C27—C28	1.405 (3)
C9—C10	1.360 (5)	C28—C29	1.369 (3)
C9—C14	1.398 (5)	C28—H28	0.9300
C10—C11	1.365 (4)	C29—C30	1.410 (3)
C10—H10	0.9300	C29—H29	0.9300
C11—C12	1.400 (3)	C30—N4	1.376 (2)
C11—H11	0.9300	C30—C31	1.402 (3)
C12—N2	1.369 (3)	C31—C32	1.374 (3)
C12—C13	1.396 (3)	C31—H31	0.9300
C13—C14	1.382 (4)	C32—H32	0.9300
C13—H13	0.9300	C33—N4	1.458 (3)
C14—H14	0.9300	C33—C34	1.509 (3)
C15—N2	1.446 (3)	C33—H33A	0.9700
C15—C16	1.511 (4)	C33—H33B	0.9700
C15—H15A	0.9700	C34—H34A	0.9600
C15—H15B	0.9700	C34—H34B	0.9600
C16—H16A	0.9600	C34—H34C	0.9600
C16—H16B	0.9600	C35—N4	1.450 (3)
C16—H16C	0.9600	C35—C36	1.516 (3)
C17—N2	1.444 (4)	C35—H35A	0.9700
C17—C18	1.562 (8)	C35—H35B	0.9700
C17—H17A	0.9700	C36—O2	1.415 (3)
C17—H17B	0.9700	C36—H36A	0.9700
C18—O1	1.468 (11)	C36—H36B	0.9700
C18—H18A	0.9700	O2—H2C	0.9600
C18—H18B	0.9700	N5—O5	1.200 (3)
O1—H1E	0.9600	N5—O4	1.217 (3)
O1—H1'2	0.8213	N5—O3	1.230 (3)
C18'—C17'	1.343 (15)	N6—O7	1.201 (3)
C18'—O1'	1.552 (19)	N6—O8	1.219 (3)
C18'—H18C	0.9700	N6—O6	1.226 (3)
C18'—H18D	0.9700	O9—H9B	0.9240
C17'—N2	1.735 (13)	O9—H9D	0.9446
C17'—H17C	0.9700		
			
N1—C1—H1A	109.5	C18'—O1'—H1E	119.6
N1—C1—H1B	109.5	C18'—O1'—H1'2	113.4
H1A—C1—H1B	109.5	H1E—O1'—H1'2	59.7
N1—C1—H1C	109.5	N3—C19—H19A	109.5
H1A—C1—H1C	109.5	N3—C19—H19B	109.5
H1B—C1—H1C	109.5	H19A—C19—H19B	109.5
N1—C2—C3	120.3 (2)	N3—C19—H19C	109.5
N1—C2—H2	119.9	H19A—C19—H19C	109.5
C3—C2—H2	119.9	H19B—C19—H19C	109.5
C2—C3—C4	121.8 (3)	N3—C20—C21	120.86 (19)
C2—C3—H3	119.1	N3—C20—H20	119.6
C4—C3—H3	119.1	C21—C20—H20	119.6
C5—C4—C3	115.4 (2)	C20—C21—C22	121.1 (2)
C5—C4—C7	106.6 (3)	C20—C21—H21	119.4
C3—C4—C7	138.0 (3)	C22—C21—H21	119.4
C5—C4—C7'	143.8 (3)	C23—C22—C21	116.14 (18)
C3—C4—C7'	100.8 (3)	C23—C22—C25	123.71 (18)
C7—C4—C7'	37.2 (2)	C21—C22—C25	120.15 (19)
C6—C5—C4	121.7 (3)	C24—C23—C22	120.6 (2)
C6—C5—H5	119.2	C24—C23—H23	119.7
C4—C5—H5	119.2	C22—C23—H23	119.7
N1—C6—C5	121.3 (3)	N3—C24—C23	121.2 (2)
N1—C6—H6	119.3	N3—C24—H24	119.4
C5—C6—H6	119.3	C23—C24—H24	119.4
C8—C7—C4	116.3 (6)	C26—C25—C22	124.25 (19)
C8—C7—H7	121.8	C26—C25—H25	117.9
C4—C7—H7	121.8	C22—C25—H25	117.9
C7—C8—C9	118.2 (6)	C25—C26—C27	128.0 (2)
C7—C8—H8	120.9	C25—C26—H26	116.0
C9—C8—H8	120.9	C27—C26—H26	116.0
C8'—C7'—C4	114.6 (7)	C32—C27—C28	116.16 (17)
C8'—C7'—H7'	122.7	C32—C27—C26	120.12 (19)
C4—C7'—H7'	122.7	C28—C27—C26	123.72 (19)
C7'—C8'—C9	113.4 (7)	C29—C28—C27	122.16 (19)
C7'—C8'—H8'	123.3	C29—C28—H28	118.9
C9—C8'—H8'	123.3	C27—C28—H28	118.9
C10—C9—C14	115.3 (2)	C28—C29—C30	121.24 (19)
C10—C9—C8	108.3 (3)	C28—C29—H29	119.4
C14—C9—C8	136.3 (4)	C30—C29—H29	119.4
C10—C9—C8'	144.9 (4)	N4—C30—C31	121.85 (18)
C14—C9—C8'	99.7 (4)	N4—C30—C29	121.51 (18)
C8—C9—C8'	36.6 (2)	C31—C30—C29	116.63 (17)
C9—C10—C11	123.7 (3)	C32—C31—C30	121.19 (19)
C9—C10—H10	118.2	C32—C31—H31	119.4
C11—C10—H10	118.2	C30—C31—H31	119.4
C10—C11—C12	121.1 (3)	C31—C32—C27	122.6 (2)
C10—C11—H11	119.5	C31—C32—H32	118.7
C12—C11—H11	119.5	C27—C32—H32	118.7
N2—C12—C13	121.9 (2)	N4—C33—C34	113.5 (2)
N2—C12—C11	121.3 (2)	N4—C33—H33A	108.9
C13—C12—C11	116.8 (2)	C34—C33—H33A	108.9
C14—C13—C12	119.9 (3)	N4—C33—H33B	108.9
C14—C13—H13	120.0	C34—C33—H33B	108.9
C12—C13—H13	120.0	H33A—C33—H33B	107.7
C13—C14—C9	123.2 (3)	C33—C34—H34A	109.5
C13—C14—H14	118.4	C33—C34—H34B	109.5
C9—C14—H14	118.4	H34A—C34—H34B	109.5
N2—C15—C16	113.4 (2)	C33—C34—H34C	109.5
N2—C15—H15A	108.9	H34A—C34—H34C	109.5
C16—C15—H15A	108.9	H34B—C34—H34C	109.5
N2—C15—H15B	108.9	N4—C35—C36	113.20 (19)
C16—C15—H15B	108.9	N4—C35—H35A	108.9
H15A—C15—H15B	107.7	C36—C35—H35A	108.9
C15—C16—H16A	109.5	N4—C35—H35B	108.9
C15—C16—H16B	109.5	C36—C35—H35B	108.9
H16A—C16—H16B	109.5	H35A—C35—H35B	107.8
C15—C16—H16C	109.5	O2—C36—C35	111.1 (2)
H16A—C16—H16C	109.5	O2—C36—H36A	109.4
H16B—C16—H16C	109.5	C35—C36—H36A	109.4
N2—C17—C18	109.7 (4)	O2—C36—H36B	109.4
N2—C17—H17A	109.7	C35—C36—H36B	109.4
C18—C17—H17A	109.7	H36A—C36—H36B	108.0
N2—C17—H17B	109.7	C2—N1—C6	119.5 (2)
C18—C17—H17B	109.7	C2—N1—C1	120.3 (2)
H17A—C17—H17B	108.2	C6—N1—C1	120.2 (2)
O1—C18—C17	107.2 (7)	C12—N2—C15	121.8 (2)
O1—C18—H18A	110.3	C12—N2—C17	119.3 (2)
C17—C18—H18A	110.3	C15—N2—C17	114.3 (2)
O1—C18—H18B	110.3	C12—N2—C17'	119.1 (4)
C17—C18—H18B	110.3	C15—N2—C17'	114.2 (3)
H18A—C18—H18B	108.5	C17—N2—C17'	44.7 (4)
C18—O1—H1E	110.4	C24—N3—C20	119.93 (17)
C18—O1—H1'2	164.5	C24—N3—C19	119.88 (19)
H1E—O1—H1'2	67.0	C20—N3—C19	120.17 (18)
C17'—C18'—O1'	94.0 (12)	C30—N4—C35	121.94 (17)
C17'—C18'—H18C	112.9	C30—N4—C33	121.17 (18)
O1'—C18'—H18C	112.9	C35—N4—C33	116.75 (17)
C17'—C18'—H18D	112.9	C36—O2—H2C	109.0
O1'—C18'—H18D	112.9	O5—N5—O4	124.8 (3)
H18C—C18'—H18D	110.3	O5—N5—O3	118.0 (3)
C18'—C17'—N2	96.2 (10)	O4—N5—O3	116.8 (2)
C18'—C17'—H17C	112.5	O7—N6—O8	121.0 (3)
N2—C17'—H17C	112.5	O7—N6—O6	120.7 (3)
C18'—C17'—H17D	112.5	O8—N6—O6	118.3 (3)
N2—C17'—H17D	112.5	H9B—O9—H9D	112.3
H17C—C17'—H17D	110.0		

### Hydrogen-bond geometry (Å, º)

**Table:**

*D*—H···*A*	*D*—H	H···*A*	*D*···*A*	*D*—H···*A*
O1—H1*E*···O2^i^	0.96	1.83	2.719 (10)	153
O2—H2*C*···O6^ii^	0.96	2.17	2.939 (4)	137
O2—H2*C*···O7^ii^	0.96	2.38	3.242 (3)	150
O9—H9*B*···O3	0.92	2.15	2.988 (4)	151
O9—H9*B*···O4	0.92	2.43	3.214 (4)	142
O9—H9*D*···O7^ii^	0.94	2.28	3.212 (4)	170
C1—H1*B*···O4^iii^	0.96	2.56	3.480 (4)	159
C2—H2···O4^iii^	0.93	2.54	3.380 (3)	151
C6—H6···O5^iv^	0.93	2.47	3.204 (4)	136
C15—H15*B*···O7^ii^	0.97	2.55	3.504 (4)	168
C19—H19*A*···O8^iv^	0.96	2.48	3.166 (3)	129
C20—H20···O5^iv^	0.93	2.47	3.348 (3)	156
C24—H24···O9^v^	0.93	2.58	3.215 (4)	126
C33—H33*A*···O1^vi^	0.97	2.53	3.400 (10)	149

Symmetry codes: (i) −*x*+1, *y*−1/2, −*z*+1/2; (ii) *x*, −*y*+1/2, *z*−1/2; (iii) *x*, −*y*+1/2, *z*+1/2; (iv) −*x*+2, −*y*+1, −*z*+2; (v) *x*, −*y*+3/2, *z*+1/2; (vi) *x*, *y*+1, *z*.
