# Supplementary figures and images for: Crystal structure of (E)-4-{4-[eth­yl(2-hydroxy­eth­yl)amino]­styr­yl}-1-methyl­pyridinium nitrate hemihydrate
Source: Acta Crystallogr E Crystallogr Commun. 2015 Jan 14;71(Pt 2):o111–2. doi: 10.1107/S2056989015000067 (PMC4384630; doi:10.1107/S2056989015000067)

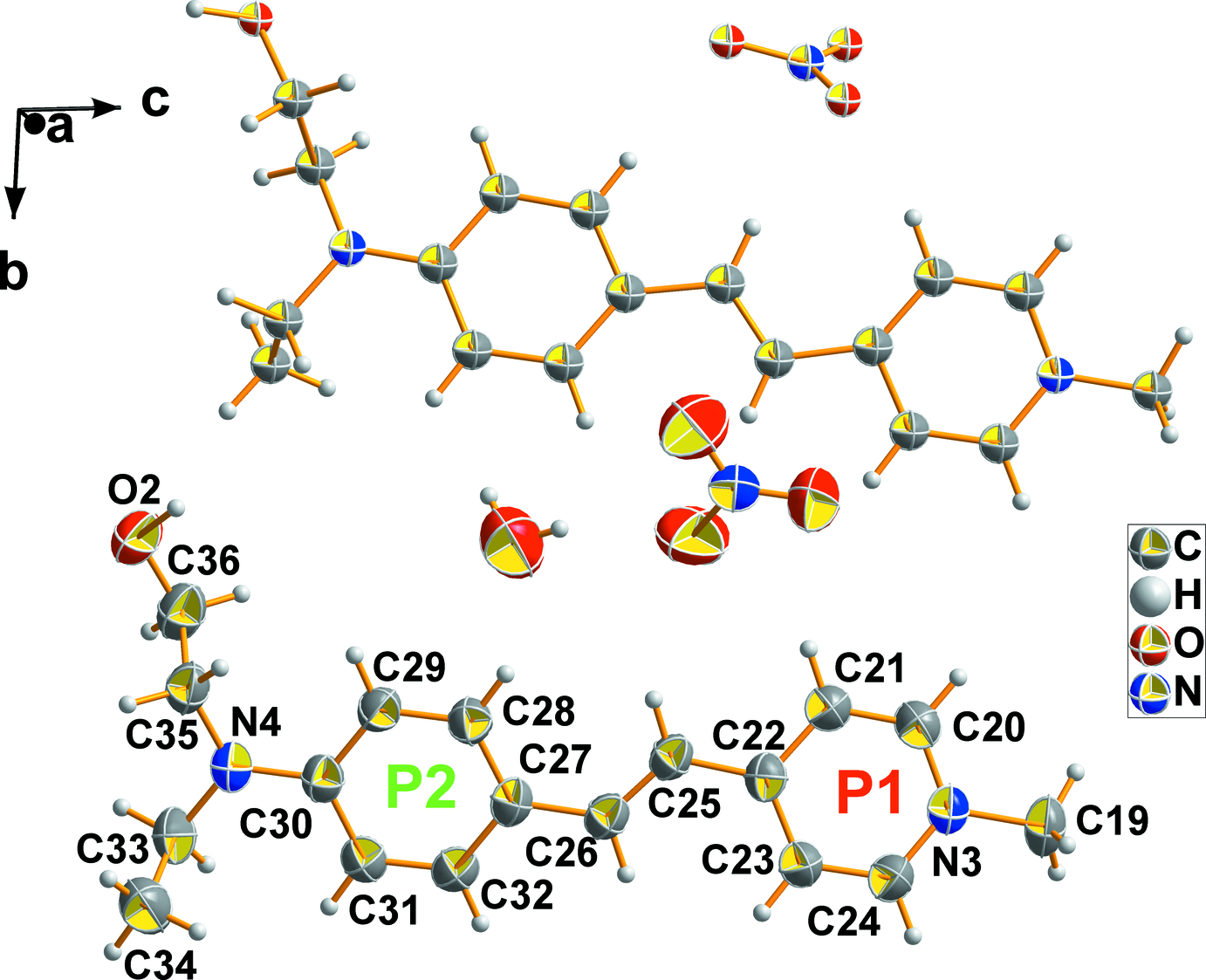

Supplement: Supplementary file 4 [file e-71-0o111-fig1.tif]
